# Effect of Donor Nb(V) Doping on the Surface Reactivity, Electrical, Optical and Photocatalytic Properties of Nanocrystalline TiO_2_

**DOI:** 10.3390/ma17020375

**Published:** 2024-01-11

**Authors:** Dmitriy Kuranov, Anastasia Grebenkina, Alexandra Bogdanova, Vadim Platonov, Sergey Polomoshnov, Valeriy Krivetskiy, Marina Rumyantseva

**Affiliations:** 1 Chemistry Department, Lomonosov Moscow State University, 119991 Moscow, Russiavkrivetsky@inorg.chem.msu.ru (V.K.); 2 Scientific-Manufacturing Complex Technological Centre, 124498 Moscow, Russia

**Keywords:** titanium dioxide, doping, pyrolysis, niobium, semiconductors, photocatalysis, optical properties, surface activity, acetone

## Abstract

In this work, we primarily aimed to study the Nb(V) doping effect on the surface activity and optical and electrical properties of nanocrystalline TiO_2_ obtained through flame-spray pyrolysis. Materials were characterized using X-ray diffraction, Raman spectroscopy and IR, UV and visible spectroscopy. The mechanism of surface reaction with acetone was studied using in situ DRIFTs. It was found that the TiO_2_-Nb-4 material demonstrated a higher conversion of acetone at a temperature of 300 °C than pure TiO_2_, which was due to the presence of more active forms of chemisorbed oxygen, as well as higher Lewis acidity of the surface. Conduction activation energies (E_act_) were calculated for thin films based on TiO_2_-Nb materials. The results of the MB photobleaching experiment showed a non-monotonic change in the photocatalytic properties of materials with an increase in Nb(V) content, which was caused by a combination of factors, such as specific surface area, phase composition, concentration of charge carriers as well as their recombination due to lattice point defects.

## 1. Introduction

Semiconductor nanocrystalline metal oxides are widely used as materials for catalysis. These can either be materials for accelerating the chemical processes of synthesis or decomposition [1,2] or materials for photobleaching dyes and purifying water following microbial contamination [3,4]. Among advanced oxidation processes, the photocatalytic processes are eco-friendly, inexpensive, long-lived and pollution-free [5]. Titanium dioxide has a suitable band structure for photocatalysis, but its efficiency is limited by the optical band gap (3.1–3.3 eV), which allows for using only about 4% of the intensity of sunlight radiation. The quantum yield of photoconversion with TiO_2_ is considered to be small and attempts have been made at its improvement given the high degree of charge carrier recombination and small specific surface [6]. Recent review articles emphasize the possibility of TiO_2_ usage for photocatalytic water splitting, wastewater treatment, air treatment and disinfection [5]. Moreover, in [7], an improved two-dimensional composite based on TiO_2_/MoS_2_ showed excellent results in the photodegradation of tetracycline, a persistent antibiotic released into wastewater from livestock production, thanks to its excellent adsorption abilities, large surface areas and effective charge-transfer ability.

Cation doping can promote the formation of suitable levels in the band gap of materials, thus helping to solve the above-mentioned problem. This can increase both the concentration of free charge carriers and oxygen vacancies, which can be used to solve difficulties in working with TiO_2_ from a catalytic point of view [8,9]. The TiO_2_-Nb system is often discussed in the scientific literature because Nb(V) has a similar ionic radius to Ti(IV), forming substitutional solid solutions in the range of up to 4 mole percent [10,11].

There are a number of techniques for the synthesis of nanosized semiconductor materials, such as sol–gel [12,13], solid-phase synthesis [14], a chemical method with calcination [15], flame-spray pyrolysis [16,17,18], etc. With the latter, it is possible to combine the processes of synthesis and doping, as well as obtain particles with a spherical morphology and narrow grain size distribution and achieve a uniform distribution of the dopant in the volume and on the surface of the material [17].

Turning to literary sources, one can highlight both well-studied points and questions left unanswered or mutually contradictory data. It has been shown that with an increase in Nb content up to 5%, there is a decrease in resistivity from 5850 to 630 µΩcm, an increase in carrier density from 6.5 to 130·10^19^ cm^−3^ as well as a decrease in charge carrier mobility from 16.3 to 7.5 cm^2^V^−1^s^−1^ [19]. An increase in the concentration of surface oxygen vacancies, which are adsorption centers, as well as a quantitative and qualitative change in the content of surface oxygen forms involved in oxidation reactions have also been shown [17,20]. The mechanism of the oxidation reaction of VOCs on the surface of semiconductor oxides was studied using the DRIFTs method [21], and acetone oxidation in particular with pure TiO_2_ [22]. However, the results obtained in these two studies cannot be compared because the experiment was carried out under different conditions. Additionally, these and other studies do not provide information on the effect of Nb doping on surface reactivity, so this question remains open. It was shown that TiO_2_-Nb materials exhibited better photocatalytic performance under ultraviolet exposure compared to pure TiO_2_ material due to increased energy conversion efficiency [23,24,25]. However, the practical application of such material is delayed by rather diverse and sometimes contradictory reports. To date, the optimal amount of the doping component Nb has not been unambiguously stated; for example, values such as 0.1 and 2 mole percent, which differ by an order of magnitude, have been declared [26,27,28]. Both an increase [26,27] and a decrease [29] in the band gap during doping have been revealed. In some studies, Nb doping leads to a significant decrease in the photocatalytic activity of TiO_2_ for unknown reasons [30,31], though we can speculate that those reasons may be different synthesis conditions affecting the concentration of point defects, specific surface area and morphology. The effect of annealing in an oxygen-rich environment on photocatalytic properties has been reported [32]; however, the specific types of chemisorbed oxygen formed on the surface in this way have not yet been indicated.

Thus, the goal of our work was to carry out a comprehensive study of the effect of doping TiO_2_ with Nb(V) on the activity of the surface, using the DRIFTS method, and on electrical properties, as well as determine the activation energy of conductivity, optical and photocatalytic properties. The novelty of this work lies in the comprehensive approach we took to generalizing the complex of effects that occur when TiO_2_ is doped with Nb(V); for instance, this is the first study to date to provide information on the acetone conversion processes on the surface of pure and Nb(V)-doped TiO_2_ nanocrystalline materials.

## 2. Materials and Methods

Materials based on TiO_2_ containing 0, 1, 2 and 4 mol% Nb(V) were obtained through flame-spray pyrolysis. Titanium (IV) triisostearoylisopropoxide (Gelest, Morrisville, PA, USA, purity 90%) and niobium (V) 2-ethylhexanoate precursor (Gelest, 95% purity) were used as precursors. The full methodology was described previously [17]. Materials collected manually from a glass fiber filter (Sigma-Aldrich, St. Louis, MO, USA) were annealed in a furnace in an air atmosphere at 500 °C for 24 h. The Nb content was verified using the X-ray fluorescence method on an M1 Mistral micro-X-ray spectrometer (Bruker, Billerica, MA, USA) with a tube voltage of 50 kV. The phase composition of the synthesized materials was established via XRD and Raman spectroscopy. The samples were studied with X-ray diffraction using a Rigaku D/MAX 2500 diffractometer with CuK_α1+2_ radiation (λ = 1.5406 Å) in the range of 5–100° 2θ and with a step of 0.02°. The phase content ratio was determined using the Chung method [33]. Raman spectra were taken using an i-Raman Plus spectrometer (BW Tek, Plainsboro, NJ, USA) equipped with a BAC 151C microscope and 532 nm laser. IR spectra were recorded on a Frontier (Perkin-Elmer, Waltham, MA, USA) Fourier transform infrared spectrometer in the range 4000–400 cm^−1^ and with a step of 1 cm^−1^ in the transmission mode.

The specific surface area of the materials was measured with the low-temperature nitrogen adsorption method; calculations were performed using the BET model. The measurements were carried out in a single point mode on a Chemisorb 2750 instrument (Micromeritics, Norcross, GA, USA) equipped with a thermal conductivity detector.

Diffuse reflectance spectra of materials obtained on a Lambda 950 spectrophotometer (Perkin Elmer, Waltham, MA, USA) in the wavelength range from 250 nm to 2.5 μm and with a step of 1 nm were converted into absorption spectra and rearranged in the Tauc coordinates in accordance with Tauc and the Davis–Mott relation [34,35].

Thick films were obtained to determine the electrical properties of materials. A powder suspension in α-terpineol was applied as a paste on the surface of a corundum plate (2 mm × 2 mm × 0.15 mm) with platinum contacts for measuring resistance on one side and for heating on the other. Line voltage was applied after each layer to remove the solvent. The resulting films were annealed at 500 °C for 12 h. The electrical conductivity was measured in a stabilized voltage mode (U = 1 V) in a 100 mL flow-through cell.

The mechanism of interaction of the surface of pure TiO_2_ material, as well as doped TiO_2_-Nb-4, with acetone vapor (20 ppm) in dry air (RH = 0%) was studied via diffusion reflectance infrared spectroscopy (DRIFT) under in situ conditions. IR spectra were recorded on a Frontier spectrometer (Perkin Elmer, Waltham, MA, USA) with a diffuse reflection attachment and an HC900 flow gas chamber (Pike Technologies, Madison, WI, USA), with a sealed ZnSe window. The spectra were recorded in the wavenumber range of 1000–4000 cm^−1^ and with a resolution of 4 cm^−1^, over 30 scans in a flow of test gas or clean air (100 mL⋅min^−1^). A weighed portion of the sample powder = 40 mg was placed in Al_2_O_3_ crucibles with a diameter of 5 mm. Before measurement, the sample was heated in a stream of clean air at a temperature of 500 °C for 30 min to remove adsorbed impurities.

A series of experiments was performed to determine the photocatalytic properties (PC) of the synthesized materials, during which 80 mL of methylene blue (MB) solution (0.01 g⋅L^−1^) with TiO_2_ nanopowder (0.2 g L^−1^) was constantly exposed to ultraviolet light (λ = 365 nm, I = 100 mA, luminous flux intensity 20 ± 2 mWcm^−2^). Mass transfer of the solution was carried out into a cuvette to take in the spectrum (Ocean Optics 4000 spectrometer, Dunedin, FL, USA), to obtain the values of optical density, and the solution was automatically returned to a cooled quartz reactor. The flow rate of the peristaltic pump was 0.08 L⋅min^−1^. A phosphate buffer solution (pH = 7.5) was used to achieve constant pH values. The concentration of MB was calculated according to the Bouguer–Lambert–Beer law from the absorption spectra of the solution.

## 3. Results and Discussion

**Figure 1:**
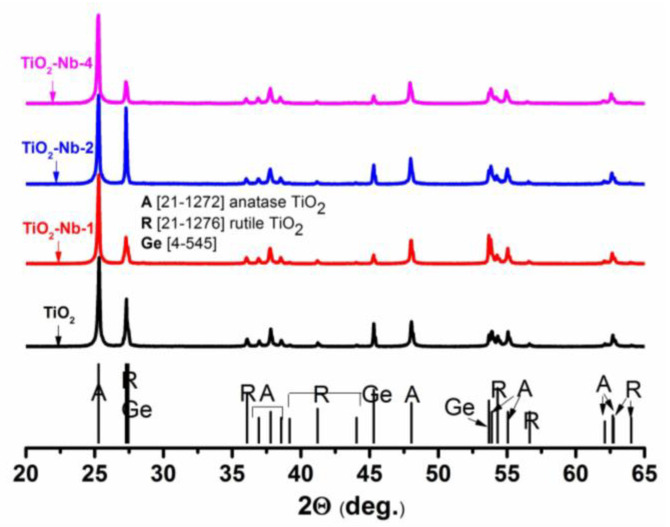
X-ray diffraction patterns of TiO_2_-Nb materials. Adapted from [17] with permission from John Wiley and Sons.

The diffraction patterns of all materials contain reflections of the anatase and rutile phases only (shown in Figure 1). The shift of the diffraction maxima occurs due to an increase in the parameters of the unit cells of the phases, due to the formation of substitutional solid solutions (the niobium cation has a larger ionic radius than the titanium cation; 0.640 and 0.605 Å, respectively). The unit cell parameters of the anatase and rutile phases were refined using several of the most intense reflections that do not overlap with reflections from the germanium standard. The obtained values correlated well with the values calculated using Vegard’s law. Linear dependences of the interplanar distances of the anatase and rutile TiO_2_ phases on the niobium content for the materials and parameters (*a*, *c*) are presented (Figure S1a–c).

The main characteristics of the materials are presented in Table 1. It is shown that niobium stabilizes the anatase phase, monotonically increases the specific surface area, and leads to an increase in the unit cell parameters [15]. The content of niobium specified during the synthesis correlates well with the real content determined using the XRF method. With an increase in the content of niobium, there is no monotonic decrease in the grain size that can be associated with an increase in the energy of the particle surface. For more detailed information, you can refer to our previous publications [17] and see the XP spectra (Figures S2 and S3 in Supplementary Materials), as well as the TEM microphotographs and histograms of the normal distribution (Figures S4 and S5 in Supplementary Materials).

**Figure 2:**
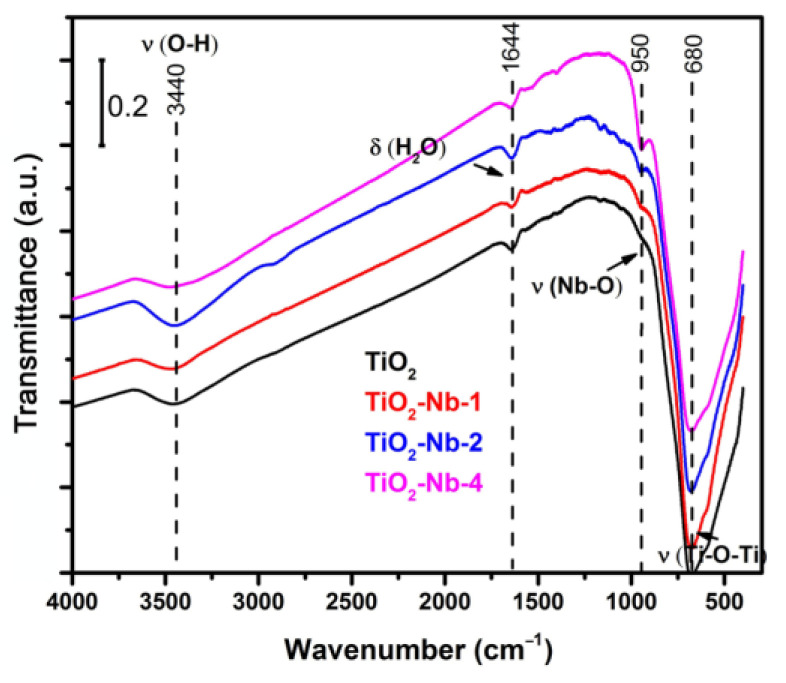
IR transmission spectra of pure and Nb-doped TiO_2_ samples.

FTIR spectra of a series of TiO_2_-Nb (0, 1, 2, 4 mol%) materials are shown in Figure 2. All spectra demonstrate a broad absorption band centered at 3440 cm^−1^, which is related to symmetric and asymmetric stretching vibrations of families of bridging hydroxyls associated via hydrogen bonds [36,37,38,39]. The presence of absorption bands at 1644 and 1540 cm^−1^ can be attributed to deformation vibrations of water molecules on the adsorption centers of the surfaces of materials such as coordinatively unsaturated titanium and niobium ions, as well as oxygen vacancies [36,37,38,39]. All spectra are dominated by a broad band at 680 cm^−1^, characteristic of TiO_2_, which is attributed to symmetric stretching vibrations of the Ti–O–Ti bridges in slightly distorted TiO_6_ octahedra linked by common vertices [40]. The intensity of the weak band at 950 cm^−1^ increases with increasing niobium content in doped materials, and its presence can be attributed to the symmetric stretching mode of O=Nb surface groups, which are present in the most strongly distorted octahedral structures [41].

**Figure 3:**
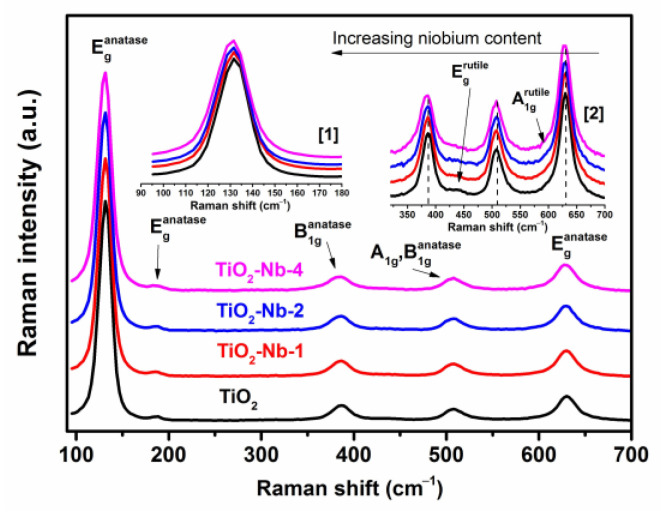
Raman spectra of pure and doped (Nb) TiO_2_ samples. Insets 1 and 2—enlarged areas of spectrum regions.

Raman spectra were recorded to confirm the phase composition and to reveal in more detail the effect of doping (Figure 3). All spectra contain the following Raman modes: E_g_ (132, 188, 631 cm^−1^), corresponding to symmetric stretching vibrations O–Ti–O; B_1g_ (~388 cm^−1^); as well as A_1g_ + B_1g_ (~508 cm^−1^), characteristic of the anatase phase [42]. Mode B_1g_ corresponds to symmetric bending vibrations O–Ti–O, and mode A_1g_ corresponds to antisymmetric bending vibrations O–Ti–O [43]. The presence of an intense mode at 132 cm^−1^ (the characteristic mode of anatase vibrations) indicates that TiO_2_ nanocrystals have a certain degree of long-range order, being well-crystallized [44,45]. The modes of the rutile phase E_g_ and A_1g_ (~506 and 615 cm^−1^, respectively) are weakly expressed due to its small fraction [46]. A red shift of the modes of the anatase phase is observed in connection with an increase in the unit cell parameters due to doping with Nb, which monotonically increases with an increase in the content of niobium in the materials (inserts 1 and 2 of Figure 3). The results of calculating the full width at half-heights of the maximum of the most intense anatase mode E_g_ confirm this conclusion: a monotonic increase is observed (17.10 cm^−1^ for TiO_2_, 17.97 cm^−1^ for TiO_2_-Nb-1, 18.21 cm^−1^ for TiO_2_-Nb-2 and 19.70 cm^−1^ for TiO_2_-Nb-4, ±0.05 cm^−1^). The redshift of the E_g_ modes during doping is a consequence of an increase in the concentration of point defects—charged oxygen vacancies [47].

### 3.1. Study of Optical Properties

**Figure 4:**
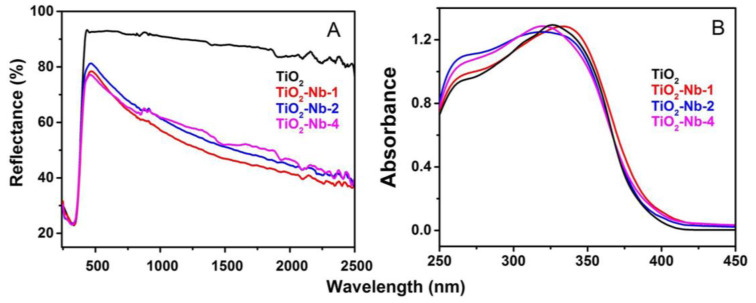
Diffuse reflectance spectra of materials (**A**). Absorption spectra in the region near the edge of the absorption band (**B**).

**Figure 5:**
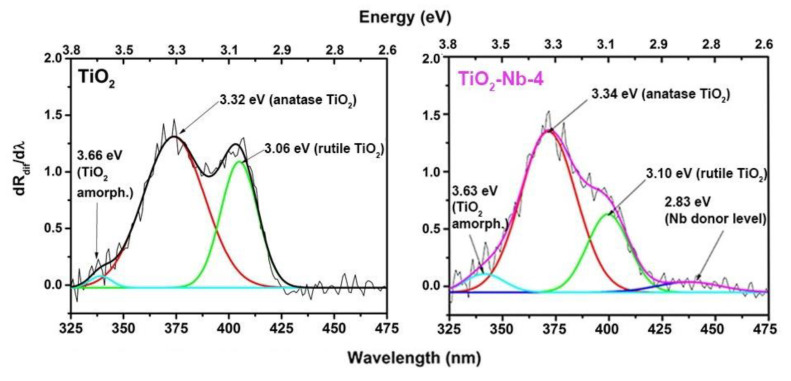
Diffuse reflectance spectra of TiO_2_ and TiO_2_-Nb-4 materials in differential form.

The diffuse reflectance spectra of the materials are presented in Figure 4 and show the average spectral reflectivity of samples in the NUV (0.25–0.4 μm), visible (0.4–0.8 μm) and NIR (0.8–2.5 μm) ranges, respectively. Undoped TiO_2_ material is highly reflective, reflecting about 90% of visible and near-infrared radiation. Doping with Nb(V) leads to an increase in absorption in the visible and IR wavelength range. The authors of [48] observed a similar phenomenon for TiO_2_ doped with Nb in the range of 1900–2700 nm, which was associated with a broad plasmon resonance peak due to free electrons. The plasmon resonance peak can have a different shape and position; we associate absorption in the IR region up to 2500 nm with absorption on free charge carriers—electrons, which is most notable for doped materials. Since the method of drawing tangents in the coordinates of the Tauc plot (Figure S6) is not entirely accurate, we also analyzed the differential diffuse reflectance spectra of the materials (Figure 5 and Figure S7) to determine the values of optical transitions. The obtained energy values of optical transitions are listed in Table 2.

It was found that doping does not affect the band gaps of the phases. The values of two optical transitions with energies of approximately 3.10 eV and 3.33 eV obtained regardless of the analysis method used can be attributed to the band gap of TiO_2_ phases, which refers to the indirect transitions allowed from the edge of the Brillouin zone to its center, namely X_1α_/X_1b_ → Γ_1b_ and Γ_3_ → X_1b_, respectively, of the rutile and anatase phases of TiO_2_ [49,50]. The weakly expressed extra peak in the region of 3.6 eV the authors of the [51] associate with the presence of a minor amount of amorphous phase. In addition to the fundamental value of the edge of the absorption band, an extra peak can be detected in the visible light reflection for the Nb-doped TiO_2_ samples. We attribute this to the appearance of a niobium cation at Ti-site ${Nb}_{Ti}^{\cdot }$ [52]. Khomenko et al. [53] also observed weak absorption bands between 2.3 eV and 2.9 eV, and they assigned them to *d*–*d* transitions associated with Ti^3+^ localized states.

As was demonstrated in our previous article [20], during XRD and paramagnetic resonance spectroscopy, Nb(V) doping leads to the formation of substitution-type solid solutions according to the quasi-chemical Equation (1):

(1) $${Nb}_{2}O_{5}\overset{{TiO}_{2}}{\to }2{Nb}_{Ti}^{\cdot }+2e^{’}+4O_{O}^{\times }+\frac{1}{2}O_{2}$$

So, the position of the energy donor level associated with Nb(V) doping can be established as 2.83 ± 0.05 eV relative to the valence band top.

### 3.2. Study of Electrical Properties

**Figure 6:**
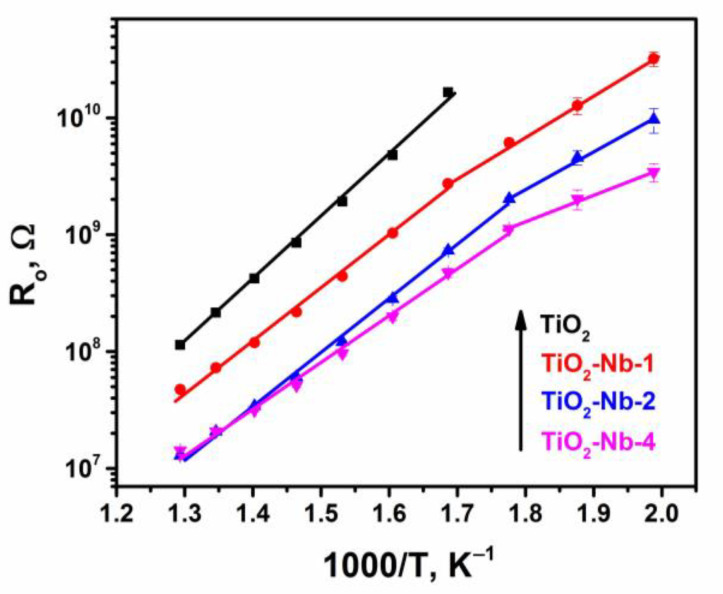
Dependences of the resistance value of materials in air on temperature in linear coordinates.

The temperature dependences of the resistance of thick TiO_2_-Nb films were obtained in the temperature range of 200–500 °C (Figure 6).

Despite the abovementioned formation of a significant number of acceptor defects during doping, the resistance of materials decreases upon Nb introduction. The temperature dependence of the resistance of pure TiO_2_ is linearized in lgR vs. 1/T coordinates, which indicates the activation character of the conductivity. The value of the activation energy of the conductivity, determined in the range of 300–500 °C, is 0.9 ± 0.03 eV, which is somewhat higher than the value determined in [54], which was 0.65 eV for an undoped thin film. The difference is due to a different phase ratio and synthesis method (Table 1). For TiO_2_-Nb samples, two linear parts can be distinguished in the high-temperature (280–500 °C) and low-temperature (200–280 °C) ranges (Figure 6). An increase in the niobium content leads to a decrease in the activation energy of conductivity in both temperature ranges (Table 3).

This effect can be explained using a model of an inhomogeneous semiconductor with large-scale potential fluctuations, which consists of smooth bands’ bending with the formation of random potential barriers [53,54]. The movement of an electron is impossible in the modulated zone if its energy is below the mobility threshold (percolation level), which is determined by the potential relief. Thus, the energy determined from the temperature dependences of conductivity is the activation energy of carriers from the Fermi level to the mobility threshold level. The concentration of charge carriers during Nb(V) doping increases; that leads to an increase in the Fermi level position and, accordingly, to a decrease in the activation energy, which is manifested in the low-temperature part of the activation dependence.

The gap between the conduction band and the states of oxygen vacancies determined in [55] was 0.75–1.18 eV for partially reduced TiO_2_. Oxygen vacancy states in anatase TiO_2_ were determined to be located 2.02–2.45 eV above the valence band, which is consistent with the data in [54] and ours (0.9 eV for undoped TiO_2_). As the niobium concentration increases, the activation energy decreases monotonically to 0.4 eV for the TiO_2_-Nb-4 material due to the formation of donor levels. In this case, there is no switching from the semiconductor type of conductivity to the metallic one due to the formation of charge traps (oxygen vacancies that localize conduction electrons on themselves), as we established in a previous article reporting our EPR study [20].

### 3.3. Conducting a Photocatalytic Experiment

**Figure 7:**
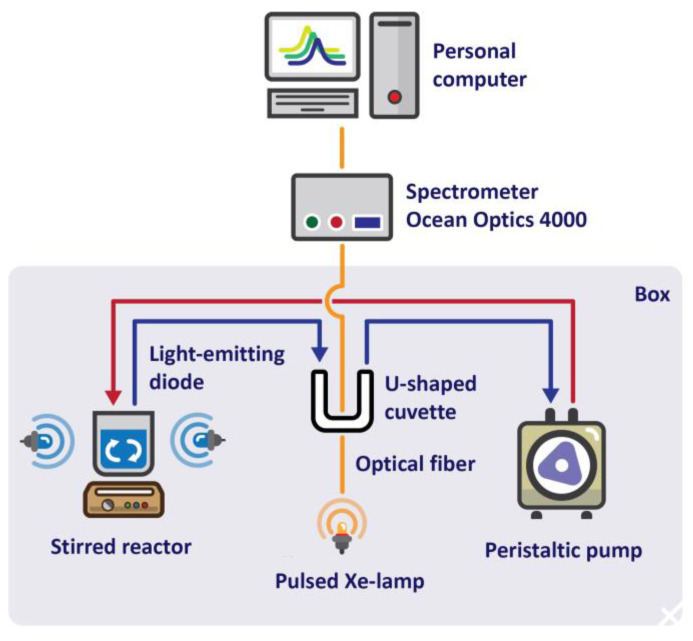
Scheme of the setup for conducting a photocatalytic experiment.

Photocatalytic decolorization of methylene blue was carried out using the setup shown in Figure 7.

**Figure 8:**
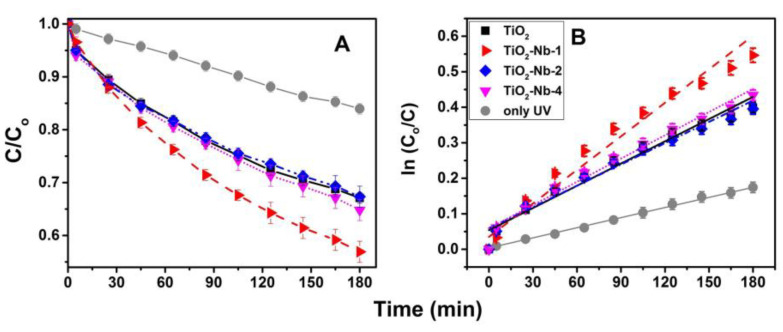
(**A**) Kinetic curves of methylene blue photobleaching in the presence of pure TiO_2_ and Nb−doped TiO_2_ photocatalysts, (**B**) same curves plotted in logarithmic coordinates.

The absorption spectrum (Figure S8) contains a band at 660–670 nm (the coordinate may vary depending on the pH of the solution) corresponding to the n-π* transition (n is a free doublet at the nitrogen atom of the C=N bond and a free doublet of the S atom at the S=C bond). Figure 8A shows the photodegradation curves, from which it follows that MB weakly photodegrades even without a photocatalyst. The introduction of pure TiO_2_ accelerates the process. It is shown that the introduction of niobium leads to a significant increase in the photocatalytic properties of TiO_2_-Nb-1 and slight increases in those of TiO_2_-Nb-2 and TiO_2_-Nb-4. In order to give a more accurate assessment of the kinetics of the processes, photodegradation curves were plotted (Figure 8B) with the coordinates ln(C_o_/C) and time, for linearization, assuming that these reactions are of the first order, and the kinetic characteristics listed in Table 4 were calculated.

It follows that the TiO_2_-Nb-1 material exhibits the highest photocatalytic activity, speeding up the photodegradation process by three times. First, this directly follows from the optical properties of the material—the appearance of a donor level, as well as the highest absorption in the IR region, indicating absorption on free charge carriers involved in the photocatalysis process. Secondly, the nonmonotonicity of the change in the PC activity with an increase in the niobium content indicates that such an important characteristic as the specific surface area plays a minor role in increasing the PC properties of materials. Thirdly, based on the data of Raman spectroscopy, the red shift of E_g_ modes upon doping is a consequence of an increase in the concentration of point defects—charged oxygen vacancies. Additional conduction electrons are also formed, reducing electrical resistance to maintain overall electrical neutrality. It can be assumed that the electron traps formed during doping will contribute to a decrease in the PC activity, as evidenced by the low PC characteristics of the TiO_2_-Nb-2 and TiO_2_-Nb-4 materials. Comparative characterization of the photocatalytic properties of doped TiO_2_ is shown in Table 5. This reveals that materials based on Nb(V)-doped TiO_2_ discussed in our article are not inferior in photocatalytic parameters to those mentioned in other works.

**Figure 9:**
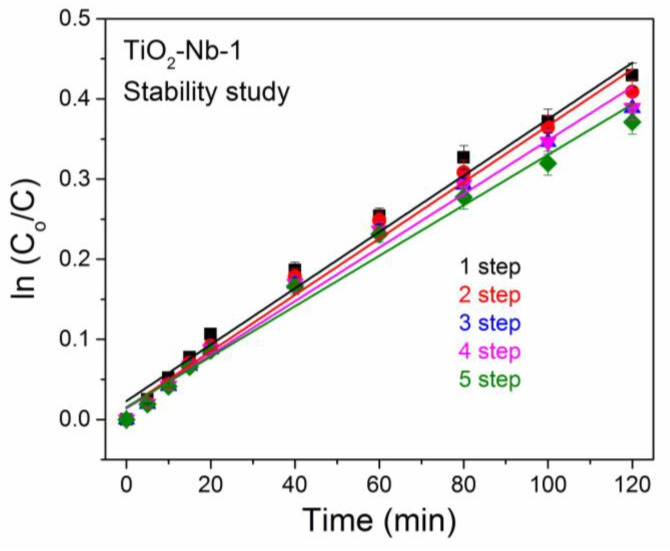
Comparison of TiO_2_-Nb-1 photocatalytic activity in 5 consecutive photobleaching cycles.

For a stability study (Figure 9), the catalyst precipitate was separated from the solution via centrifugation at the end of each cycle, washed with buffer solution and used in subsequent cycles. The results demonstrate that Nb(V)-doped TiO_2_ does not lose its photocatalytic activity after five successive cycles of methylene blue photobleaching. A slight decrease in photocatalytic activity may be due to loss of catalyst during washing and the adhesion of dye molecules on the surface of the photocatalyst. To improve the stability of Nb(V)-doped TiO_2_ photocatalytic activity, it is recommended that the catalyst should be washed with alcohol or acetone rather than with water or it should be annealed in a furnace.

### 3.4. Study of Catalytic Properties of Materials

In our previous work [20], it was found that an increase in the Nb(V) concentration leads to an increase in the TiO_2_ sensor response when detecting the volatile organic compounds (VOCs), especially in the case of acetone. To determine the cause of this effect, the processes responsible for the sensor response during the interaction of pure TiO_2_ and TiO_2_-Nb4 were investigated in situ using the DRIFT method.

**Figure 10:**
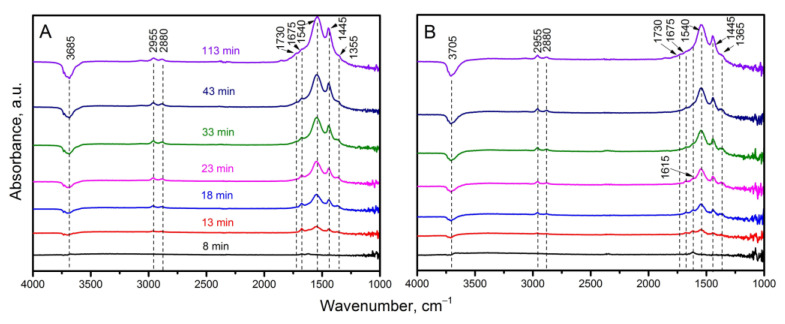
In situ DRIFT spectra taken during signal accumulation (adsorption of 20 ppm acetone and surface reaction processes) (**A**) for pure and (**B**) modified TiO_2_-Nb-4.

Absorption spectra taken during exposure to acetone vapor, reflecting ongoing adsorption processes and its chemical transformations on the surface of pure TiO_2_ and TiO_2_-Nb-4, are shown in Figure 10.

First of all, the adsorption of acetone is realized by passing its vapor over the same catalytic and adsorption centers of the material surface for which OH groups compete (oxygen vacancies, coordinatively unsaturated metal cations), in accordance with Equation (2):


(2) $${\mathrm{CH}}_{3}{\mathrm{COCH}}_{3}\to {\mathrm{CH}}_{3}{\mathrm{COCH}}_{3(\mathrm{ads})}$$

This is confirmed by a monotonous decrease in absorption in the spectral region of 3400–3900 cm^−1^ υ (OH) during the experiment and the appearance of a weak band with a frequency of 1675 cm^−1^, which corresponds to the stretching vibration of the C=O bond, bands υ(C-H) at 2880 and 2955 cm^−1^, as well as δ (CH_3_) at 1445 cm^−1^ of acetone adsorbed in molecular form [37]. Since the selected temperature T = 300 °C is the condition for the formation of the maximum sensor response for the previously obtained dependences S(T) (see [20]), in the spectra (Figure 9), we can observe the appearance of bond bands corresponding to the products of the surface reaction of acetone with chemisorbed forms of oxygen. This reaction causes a change in the electrical resistance of materials, such as υ_as_ and υ_s_ (COO^−^) at 1540 and 1355 cm^−1^, respectively, and δ(H_2_O) at 1615 cm^−1^ [37]. Weak absorption bands at *k* = 1855, 1780 and 1730 cm^−1^ are clearly related to adsorbed acetone intermediates [22,37,60,61,62].

According to [63], as well as [37], absorption bands in this region can be attributed to symmetrical and asymmetrical stretching vibrations of the C=O group of anhydrides. Based on the ratio of the intensities of the bands 1855 (weak, symmetric vibration) and 1730 cm^−1^ (stronger, asymmetric vibration), it can be assumed that the anhydride is present in the cyclic form [63]. There are catalytic centers on the surface of titanium dioxide, and at elevated temperatures, processes of aldol condensation, acetylation, cyclization, dehydrogenation and dehydration of such a chemically active molecule as acetone can occur on them. These bands are weak, and, therefore, they reflect the occurrence of a parallel, non-predominant course of the reaction. The main path is oxidation through the formation of acetates with their further complete oxidation to carbon dioxide and water, which is consistent with previous results [21].

It should be mentioned that in [22], the process of formation of the intermediate mesityl oxide under standard conditions (room temperature) on the surface of titanium dioxide, as well as in solution with the participation of a catalyst containing a catalytic Al^3+^ center, was considered [61,62], and convincing evidence was given. However, in our case, the formation of this molecule did not occur, probably since there was a discrepancy between the absorption bands of the (tabular) groups of mesityl oxide and the absorption bands in our spectra.

**Figure 11:**
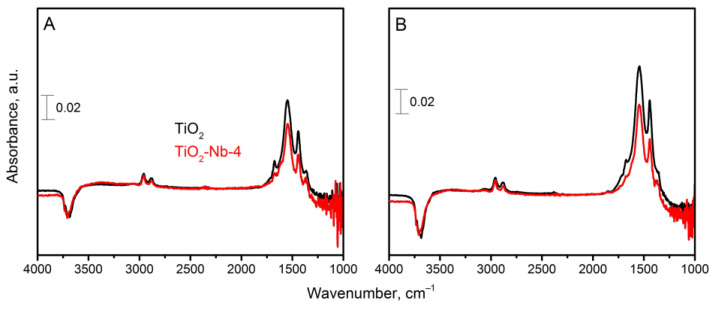
In situ DRIFT spectra for pure and modified TiO_2_-Nb-4 taken during signal accumulation ((**A**)—23 min and (**B**)—43 min since the beginning of the experiment).

It seems possible to conduct a comparative analysis of the intensities of the IR profile bands of the TiO_2_ and TiO_2_-Nb-4 materials since the experimental conditions were identical. Selected comparative absorption spectra for the two materials are shown in Figure 11.

It was shown that signal accumulation for pure TiO_2_ material occurred relatively faster than for the TiO_2_-Nb-4 material, which was associated with the surface reactivity and conversion degree.

**Figure 12:**
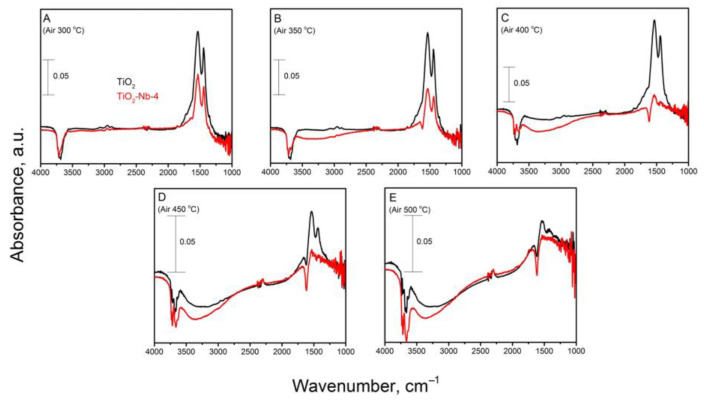
In situ DRIFT spectra of TiO_2_ and TiO_2_-Nb-4 taken during the passage of clean air at RH = 0% and T = 300–500 °C (**A**–**E**).

Absorption spectra taken during exposure to clean air (RH = 0%) in order to release adsorption centers are presented in Figure 12.

We sought to achieve the most complete removal of intermediates from the adsorption centers on the surface of materials during this experiment; therefore, we carried out the process in a dynamic temperature regime, gradually heating the materials to 500 °C (temperature step, 50 °C; holding time for one stage, 20 min). The appearance of additional broadened absorption bands was clear (for example, in the region of *k* = 3000–3500 cm^−1^). This was associated with the transfer of charge carriers (electrons from the valence band to the conduction band), which received additional energy with increasing temperature.

Partial oxidation products and adsorbed intermediates on the surface of pure TiO_2_ are most completely removed from adsorption centers at temperatures above T = 400 °C, as evidenced by a significant decrease in absorption in the region *k* = 1730–1855 cm^−1^. The maximum possible desorption (conversion) is realized at T = 500 °C. The splitting of the absorption band in the region *k* = 3500–3800 cm^−1^ into several bands indicates the presence of different types of OH groups on the surface of materials—terminal (closer to 3700 cm^−1^), bridging (bands are shifted to lower wave numbers) and others. In support of this, previous articles [64,65] claim that at least 12 types of hydroxyl groups exist on the surface of titanium dioxide.

**Figure 13:**
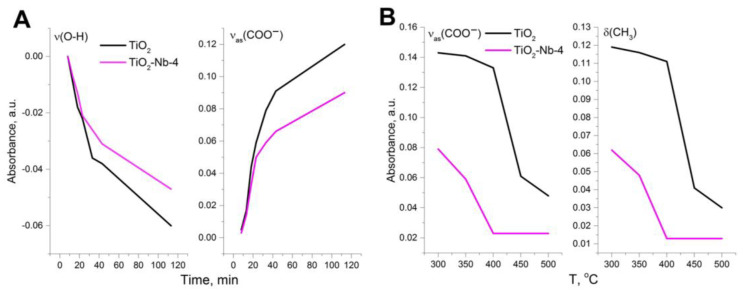
Dynamics of changes in the absorption of bands of (**A**) OH^−^ and COO^−^ groups upon exposure to 20 ppm acetone (T = 300 °C, RH = 0%); (**B**) CH_3_ and COO^−^ groups during exposure to clean air (RH = 0%, duration of one temperature stage = 30 min).

For greater clarity, the combined data in Figure 9, Figure 10 and Figure 11, reflecting the dynamics of changes in the absorption of bands attributed to vibrations of the bonds of the OH and COO^−^ groups, are presented in Figure 13.

The absorption of the acetate group-band increases faster for pure TiO_2_ material than for TiO_2_-Nb-4 material at the stage of signal accumulation (adsorption) and decreases for OH^−^ groups. If this process were realized at room temperature, or temperatures much lower than the temperature at which the sensory signal occurs, then an assumption would be made about the number of adsorption sites for the material. In this case, this is not a consequence of more efficient adsorption of acetone on the surface due to the high temperature of the experiment, and the surface reaction for the TiO_2_-Nb-4 material with chemisorbed oxygen occurs quite quickly. The conversion of acetone and the removal of intermediates from adsorption sites occurs much more quickly (and at a lower temperature) in the case of the TiO_2_-Nb-4 material, which will affect sensor characteristics of the material such as response/recovery time and sensor signal magnitude, as well as the optimal temperature for the formation of a sensor signal.

To discuss the results obtained, we turn to two complementary concepts. The first comes down to the fact that on the surface of a semiconductor oxide, there is always chemisorbed oxygen in one form or another, which localizes the electron density on itself. Doping can contribute to a change in the total amount of chemisorbed oxygen, as well as affect the predominant charge form of its occurrence. Previously, the authors of [17] determined the values of the coefficient *m*, which, in accordance with the theory described in [66], indicate the probabilistic presence of various forms of chemisorbed oxygen ($O^{2-}$, $O^{-}$ and $O_{2}^{-}$) on the surface of n-type semiconductor oxides. The values of the calculated coefficient *m*, obtained from the dependences of electrical conductivity on the partial pressure of oxygen, are *m* = 0.26 and 0.64, respectively, for TiO_2_ and TiO_2_-Nb-4 materials at a sensor operating temperature of 300 °C [17].

Thus, the most probable form of chemisorbed oxygen for an undoped material is $O^{2-}$, and they are $O^{-}$ and $O_{2}^{-}$ (predominantly $O^{-}$) for the TiO_2_-Nb-4 material. The $O^{2-}$ form is a less electrophilic particle, which will contribute to a less intense conversion of the target gas on the surface of the semiconductor. In addition, the same work showed a tendency for the proportion of chemisorbed oxygen to increase with increasing Nb content. The authors attributed this mainly to an increase in the specific surface area during doping.

It follows [20] that large values of the sensor signal *S* towards acetone (the formula contains the values of the resistance of the material in air and during exposure to acetone) are observed for Nb-doped material, with a tendency to shift the maximum of the sensor signal to the region of lower temperatures, which is a consequence of the oxidation reactions with more active forms $O^{-}$ and $O_{2}^{-}$ as well as the higher content of the fraction of chemisorbed oxygen in general.

On the other hand, the adsorption of acetone on the surface of semiconductor oxides depends on the Lewis acidity of the surface of the materials. The oxygen atom in the acetone molecule has lone electron pairs that tend to bind to acid sites (metal cations with vacant orbitals). The inclusion of Nb in the Ti sublattice affects the acidity of the surface, since, based on acidity theory, Nb^5+^ is more acidic than Ti^4+^. The acidity values of ions based on Sanderson’s concept of electronegativity were determined to be 3.046 and 3.895 for TiO_2_ and Nb_2_O_5_, respectively [67]. Thus, doping with niobium promotes a greater affinity of acetone for the surface of the material, which also affects the degree of conversion.

So, it should be noted that the photocatalytic activity of Nb-doped TiO_2_ in methylene blue photobleaching and the catalytic activity in acetone oxidation depend differently on the Nb(V) concentration in TiO_2_. It has been shown that Nb(V)-doping non-monotonically affects the efficiency of photocatalytic discoloration of methylene blue. In this case, the TiO_2_-Nb-1 material shows comparatively better performance. A further increase in Nb(V) concentration leads to the enhancement of acceptor defectivity and decreases the photocatalytic activity. On the contrary, in acetone conversion, TiO_2_-Nb-4 material shows the best characteristics. In this case, the determining factor is the acidity of the surface and the formation of more active forms of oxygen. These parameters have monotonic dependence on the Nb(V) content. The reproducibility of material properties has been shown in this article (stability study), as well as in our article about sensor properties [20]. We propose that materials based on niobium-doped titanium dioxide exhibit stable sensor signals and do not change their parameters even during long-term measurements.

## 4. Conclusions

This work has showed the effect of doping with niobium(V) on the electrical and optical properties of titanium dioxide, which makes this material more attractive for practical applications, for example, for photocatalysis, as was shown in this work, or for gas sensors. It has been shown through Raman and optical spectroscopy that the introduction of niobium (up to 4 mol%) into the TiO_2_ structure leads to an increase in the concentration of point defects such as oxygen vacancies. Other crystalline phases do not form during synthesis via flame-spray pyrolysis, and the grain size of the anatase and rutile TiO_2_ phases is maintained at the nanoscale level. Multiple increases in the photocatalytic properties during doping, observed for the TiO_2_-Nb-1 material, occur mainly due to the formation of donor levels in the optical band gap of the semiconductor, an increase in the specific surface area and, most importantly, release of conduction electrons. A further increase in the content of niobium does not lead to an increase in the photocatalytic properties of the material, presumably due to the formation of electron traps (oxygen vacancies of a different type), which localize conduction electrons on themselves. In addition, there is a small probability of a shielding effect arising from the presence of Nb_2_O_5_ on the surface of TiO_2_-Nb-2 and TiO_2_-Nb-4 materials in ultra-small quantities that are not visible in X-ray diffraction patterns. It has been established that the TiO_2_-Nb-4 material exhibits greater reactivity during the conversion of acetone at a temperature of 300 °C at the solid–gas interface compared to the undoped TiO_2_ material due to the presence of more active forms of chemisorbed oxygen on the surface and high surface acidity. Since the process of photocatalysis of the dye in solution and the process of oxidation of acetone in the gas phase occur via different mechanisms, in the first case, the established optimal niobium content is 1 mol% and, in the second, it is 4 mol%.

## Figures and Tables

**Table 1:** Characteristics of the materials. Adapted from [17] with permission from John Wiley and Sons.

Material	χ_XRF_ (Nb) [mol%]	χ_XPS_ (Nb) [mol%]	d_XRD_ ± 2 [nm]		Normal Particle Size Distribution (TEM, nm)	Anatase/ Rutile Ratio	S_spec_, m^2^/g	Agglomer. Degree, d_BET_/d_XRD_	O_surf_/ O_latt (XPS)_
			Anatase	Rutile					
TiO_2_	0	0	21	23	14 ± 7	6.9	34 ± 2	2.1	0.14
TiO_2_-Nb-1	1.3 ± 0.1	0.7	21	26	-	8.7	41 ± 3	1.7	0.19
TiO_2_-Nb-2	2.4 ± 0.2	1.6	20	28	-	12.0	49 ± 3	1.4	0.20
TiO_2_-Nb-4	4.7 ± 0.4	3.4	21	26	13 ± 5	12.7	66 ± 5	1.0	0.21

**Table 2:** Energies of optical transitions of materials.

Optical Transition (eV)	TiO_2_	TiO_2_-Nb-1	TiO_2_-Nb-2	TiO_2_-Nb-4
E1 (amorph)	3.66 ± 0.01	3.63 ± 0.01	3.64 ± 0.01	3.63 ± 0.01
E2 (anatase)	3.32 ± 0.02	3.33 ± 0.02	3.33 ± 0.02	3.33 ± 0.02
E3 (rutile)	3.06 ± 0.04	3.10 ± 0.04	3.11 ± 0.04	3.10 ± 0.04
E4 (donor level)	-	2.83 ± 0.05	2.83 ± 0.05	2.83 ± 0.05

**Table 3:** The values of conductivity activation energies E_act_ for two linear sections of Figure 6.

Material	E_act_, eV	
	280–500 °C	200–280 °C
TiO_2_	0.90 ± 0.03	-
TiO_2_-Nb-1	0.75 ± 0.01	0.62 ± 0.02
TiO_2_-Nb-2	0.75 ± 0.01	0.58 ± 0.01
TiO_2_-Nb-4	0.68 ± 0.03	0.40 ± 0.01

**Table 4:** Kinetic parameters of the photodegradation process.

Material	^(1)^ Slope × 10^−3^	^(2)^ K_i_/K_UV_	^(3)^ t_½_ = ln2/k_i_
only UV	0.97 ± 0.02	-	715
TiO_2_	2.08 ± 0.12	2.15	333
TiO_2_-Nb-1	2.93 ± 0.17	2.99	237
TiO_2_-Nb-2	2.02 ± 0.12	2.08	343
TiO_2_-Nb-4	2.22 ± 0.16	2.28	312

^(1)^ The slope of the dependences in the linear coordinates of Figure 8B is the rate constant of the photobleaching reaction. ^(2)^ The ratio of the reaction constants with a catalyst to the reaction constant without it shows how many times faster the reaction is. ^(3)^ Half-conversion time calculated from the reaction rate value.

**Table 5:** Comparative characterization of the photocatalytic properties of doped TiO_2_.

Material, Synthesis Method, Morphology and Concentration	K, min^−1^	Degradation Value (per Time, % min^−1^)	Stability Data	Model Dye	Ref.
TiO_2_-Nb-C, sol–gel, 30 ± 2 nm, spherical (25 mgL^−1^)	3.0–4.0 × 10^−4^	20% per 90 min^−1^	Slight reduction in the degree of photobleaching after five cycles	Rhodamine B (2 mgL^−1^)	[56]
TiO_2_-Nb-1, hydrothermal synthesis, nanorods up to 1 μm (1000 mgL^−1^)	Not mentioned	50% per 50 min^−1^	Not mentioned	Rhodamine B (20 ppm)	[57]
TiO_2_-Nb-1, coprecipitation, 9 nm, spherical (0.17 gL^−1^)	Not mentioned	70% per 60 min^−1^	Not mentioned	Methylene blue (10 µmol)	[58]
TiO_2_:1 at.% Nb, one-step FSS, 30 nm (0.4 mg cm^−3^)	2.0 × 10^−3^	20% per 200 min^−1^	Not mentioned	Methylene blue (5.13 × 10^−5^ M)	[11]
TiO_2_:5 at.% Nb, sol–gel, 500 nm agglomerates, spherical (concentration is not specified)	2.8 × 10^−3^	15% per 60 min^−1^	Not mentioned	Methyl orange (10 mgL^−1^)	[59]
TiO_2_-Nb-1, FSP, 20 ± 2 nm, spherical (0.2 gL^−1^)	2.93 ± 0.17 × 10^−3^	40% per 180 min^−1^	Slight reduction in the degree of photobleaching after five cycles	Methylene blue (0.01 gL^−1^)	This work

## Data Availability

The data presented in this study are available on request from the corresponding author.

## Supplementary Materials

The following supporting information can be downloaded at: https://www.mdpi.com/article/10.3390/ma17020375/s1, Figure S1a: The values of interplanar spaces for the family of planes 101 of TiO_2_ anatase phase during doping with Nb(V), Figure S1b: The values of interplanar spaces for the family of planes 101 of TiO_2_ rutile phase during doping with Nb(V), Figure S1c: The values of unit cell parameters of TiO_2_ anatase phase during doping with Nb(V), Figure S1d: The values of unit cell parameters of TiO_2_ rutile phase during doping with Nb(V), Figure S2: Review XP spectra of TiO_2_ and TiO_2_-Nb-4 materials, Figure S3: Detailed XP spectra of TiO_2_ materials, Figure S4: Bright-field TEM photograph and histogram of normal distribution of TiO_2_ pure material, Figure S5: Bright-field TEM photograph and histogram of normal distribution of TiO_2_-Nb-4 material, Figure S6: Tauc plot for TiO_2_-Nb-4 materials (values of transition energy are specified), Figure S7: Differential diffuse reflectance spectra of materials TiO_2_-Nb-1 and TiO_2_-Nb-2, Figure S8: Absorption spectra of methylene blue solutions taken during photocatalysis.

## References

[1] Scirè S., Fiorenza R., Bellardita M., Palmisano L., Parrino F., Palmisano L. (2021). 21—Catalytic Applications of TiO_2_. Metal Oxides.

[2] Lee V.J. (2003). Catalysis on Wide-Band-Gap Semiconductors. J. Chem. Phys..

[3] Armaković S.J., Savanović M.M., Armaković S. (2023). Titanium Dioxide as the Most Used Photocatalyst for Water Purification: An Overview. Catalysts.

[4] Li Y., Chen F., He R., Wang Y., Tang N. (2019). Semiconductor Photocatalysis for Water Purification. Nanoscale Materials in Water Purification: Micro and Nano Technologies.

[5] Hunge Y.M., Yadav A.A., Kang S.-W., Mohite B.M. (2023). Role of Nanotechnology in Photocatalysis Application. Recent Pat. Nanotechnol..

[6] Schneider J., Matsuoka M., Takeuchi M., Zhang J., Horiuchi Y., Anpo M., Bahnemann D.W. (2014). Understanding TiO_2_ Photocatalysis: Mechanisms and Materials. Chem. Rev..

[7] Hunge Y.M., Yadav A.A., Kang S.W., Kim H. (2022). Photocatalytic Degradation of Tetracycline Antibiotics Using Hydrothermally Synthesized Two-Dimensional Molybdenum Disulfide/Titanium Dioxide Composites. J. Colloid Interface Sci..

[8] Bharti B., Kumar S., Lee H.-N., Kumar R. (2016). Formation of Oxygen Vacancies and Ti^3+^ State in TiO_2_ Thin Film and Enhanced Optical Properties by Air Plasma Treatment. Sci. Rep..

[9] Kumaravel V., Rhatigan S., Mathew S., Michel M.C., Bartlett J., Nolan M., Hinder S.J., Gascó A., Ruiz-Palomar C., Hermosilla D. (2020). Mo Doped TiO_2_: Impact on Oxygen Vacancies, Anatase Phase Stability and Photocatalytic Activity. J. Phys. Mater..

[10] Teleki A., Bjelobrk N., Pratsinis S.E. (2008). Flame-Made Nb- and Cu-Doped TiO_2_ Sensors for CO and Ethanol. Sens. Actuators B Chem..

[11] Michalow K.A., Flak D., Heel A., Parlinska-Wojtan M., Rekas M., Graule T. (2012). Effect of Nb Doping on Structural, Optical and Photocatalytic Properties of Flame-Made TiO_2_ Nanopowder. Environ. Sci. Pollut. Res..

[12] Liang Y., Sun S., Deng T., Ding H., Chen W., Chen Y. (2018). The Preparation of TiO_2_ Film by the Sol-Gel Method and Evaluation of Its Self-Cleaning Property. Materials.

[13] Liu R., Ren Y., Wang J., Wang Y., Jia J., Zhao G. (2021). Preparation of Nb-Doped TiO_2_ Films by Sol-Gel Method and Their Dual-Band Electrochromic Properties. Ceram. Int..

[14] Han X., Li C., Tang P., Feng C., Yue X., Zhang W. (2022). Solid-Phase Synthesis of Titanium Dioxide Micro-Nanostructures. ACS Omega.

[15] Zaitsev S.V., Moon J., Takagi H., Awano M. (2000). Preparation and Characterization of Nanocrystalline Doped TiO_2_. Adv. Powder Technol..

[16] Tolek W., Khruechao K., Pongthawornsakun B., Mekasuwandumrong O., Aires F.J.C.S., Weerachawanasak P., Panpranot J. (2021). Flame Spray-Synthesized Pt-Co/TiO_2_ Catalysts for the Selective Hydrogenation of Furfural to Furfuryl Alcohol. Catal. Commun..

[17] Kuranov D., Platonov V., Khmelevsky N., Bozhev I., Maksimov S., Rumyantseva M., Krivetskiy V. (2022). Effect of Nb(V) Doping on the Structure and Oxygen Chemisorption on Nanocrystalline TiO_2_. ChemistrySelect.

[18] Krivetskiy V., Zamanskiy K., Beltyukov A., Asachenko A., Topchiy M., Nechaev M., Garshev A., Krotova A., Filatova D., Maslakov K. (2019). Effect of AuPd Bimetal Sensitization on Gas Sensing Performance of Nanocrystalline SnO_2_ Obtained by Single Step Flame Spray Pyrolysis. Nanomaterials.

[19] Dorow-Gerspach D., Mergel D., Wuttig M. (2021). Effects of Different Amounts of Nb Doping on Electrical, Optical and Structural Properties in Sputtered TiO_2−x_ Films. Crystals.

[20] Kuranov D., Platonov V., Konstantinova E., Grebenkina A., Rumyantseva M., Polomoshnov S., Krivetskiy V. (2023). Gas Sensing with Nb(V) Doped Nanocrystalline TiO_2_: Sensitivity and Long-Term Stability Study. Sens. Actuators B Chem..

[21] Marikutsa A., Novikova A., Rumyantseva M., Khmelevsky N., Gaskov A. (2021). Comparison of Au-Functionalized Semiconductor Metal Oxides in Sensitivity to VOC. Sens. Actuators B Chem..

[22] Alalwan H., Alminshid A. (2020). An In-Situ DRIFTS Study of Acetone Adsorption Mechanism on TiO_2_ Nanoparticles. Spectrochim. Acta Part A Mol. Biomol. Spectrosc..

[23] Kong L., Wang C., Wan F., Li L., Zhang X., Liu Y. (2017). Transparent Nb-Doped TiO_2_ Films with the [001] Preferred Orientation for Efficient Photocatalytic Oxidation Performance. Dalt. Trans..

[24] Stefik M., Heiligtag F.J., Niederberger M., Grätzel M. (2013). Improved Nonaqueous Synthesis of TiO_2_ for Dye-Sensitized Solar Cells. ACS Nano.

[25] Lü X., Mou X., Wu J., Zhang D., Zhang L., Huang F., Xu F., Huang S. (2010). Improved-Performance Dye-Sensitized Solar Cells Using Nb-Doped TiO_2_ Electrodes: Efficient Electron Injection and Transfer. Adv. Funct. Mater..

[26] Das C., Roy P., Yang M., Jha H., Schmuki P. (2011). Nb Doped TiO_2_ Nanotubes for Enhanced Photoelectrochemical Water-Splitting. Nanoscale.

[27] Yang M., Kim D., Jha H., Lee K., Paul J., Schmuki P. (2011). Nb Doping of TiO_2_ Nanotubes for an Enhanced Efficiency of Dye-Sensitized Solar Cells. Chem. Commun..

[28] Su H., Huang Y.-T., Chang Y.-H., Zhai P., Hau N.Y., Cheung P.C.H., Yeh W.-T., Wei T.-C., Feng S.-P. (2015). The Synthesis of Nb-Doped TiO_2_ Nanoparticles for Improved-Performance Dye Sensitized Solar Cells. Electrochim. Acta.

[29] Chandiran A.K., Sauvage F., Casas-Cabanas M., Comte P., Zakeeruddin S.M., Graetzel M. (2010). Doping a TiO_2_ Photoanode with Nb5+ to Enhance Transparency and Charge Collection Efficiency in Dye-Sensitized Solar Cells. J. Phys. Chem. C.

[30] Emeline A.V., Furubayashi Y., Zhang X., Jin M., Murakami T., Fujishima A. (2005). Photoelectrochemical Behavior of Nb-Doped TiO_2_ Electrodes. J. Phys. Chem. B.

[31] Yang S.-L., Wu J.-M. (1995). Effects of Nb_2_O_5_ in (Ba, Bi, Nb)-Added TiO_2_ Ceramic Varistors. J. Mater. Res..

[32] Khan S., Cho H., Kim D., Han S.S., Lee K.H., Cho S.-H., Song T., Choi H. (2017). Defect Engineering toward Strong Photocatalysis of Nb-Doped Anatase TiO_2_: Computational Predictions and Experimental Verifications. Appl. Catal. B Environ..

[33] Chung F.H. (1974). Quantitative Interpretation of X-Ray Diffraction Patterns of Mixtures. I. Matrix-Flushing Method for Quantitative Multicomponent Analysis. J. Appl. Crystallogr..

[34] Kubelka P., Munk F. (1931). A Contribution to the Optics of Pigments. Z. Tech. Phys.

[35] López R., Gómez R. (2012). Band-Gap Energy Estimation from Diffuse Reflectance Measurements on Sol–Gel and Commercial TiO_2_: A Comparative Study. J. Sol-Gel Sci. Technol..

[36] Atherton K., Newbold G., Hockey J.A. (1971). Infra-Red Spectroscopic Studies of Zinc Oxide Surfaces. Discuss. Faraday Soc..

[37] Socrates G. (2004). Infrared and Raman Characteristic Group Frequencies: Tables and Charts.

[38] Nakamoto K. (2009). Infrared and Raman Spectra of Inorganic and Coordination Compounds, Part B: Applications in Coordination, Organometallic, and Bioinorganic Chemistry.

[39] Seki T., Chiang K.-Y., Yu C.-C., Yu X., Okuno M., Hunger J., Nagata Y., Bonn M. (2020). The Bending Mode of Water: A Powerful Probe for Hydrogen Bond Structure of Aqueous Systems. J. Phys. Chem. Lett..

[40] Bansal J., Tabassum R., Swami S.K., Bishnoi S., Vashishtha P., Gupta G., Sharma S.N., Hafiz A.K. (2020). Performance Analysis of Anomalous Photocatalytic Activity of Cr-Doped TiO_2_ Nanoparticles [Cr_(*X*)_TiO_2(1 − *X*)_]. Appl. Phys. A.

[41] Stošić D., Bennici S., Pavlović V., Rakić V., Auroux A. (2014). Tuning the Acidity of Niobia: Characterization and Catalytic Activity of Nb_2_O_5_–MeO_2_ (Me = Ti, Zr, Ce) Mesoporous Mixed Oxides. Mater. Chem. Phys..

[42] Loudon R. (2001). The Raman Effect in Crystals. Adv. Phys..

[43] Verma R., Mantri B., Kumar Srivastava A. (2015). Shape Control Synthesis, Characterizations, Mechanisms and Optical Properties of Larg Scaled Metal Oxide Nanostructures of ZnO and TiO_2_. Adv. Mater. Lett..

[44] Bersani D., Lottici P.P., Ding X.-Z. (1998). Phonon Confinement Effects in the Raman Scattering by TiO_2_ Nanocrystals. Appl. Phys. Lett..

[45] Zhang W.F., Zhang M.S., Yin Z., Chen Q. (2000). Photoluminescence in Anatase Titanium Dioxide Nanocrystals. Appl. Phys. B.

[46] Mazza T., Barborini E., Piseri P., Milani P., Cattaneo D., Bassi A.L., Bottani C.E., Ducati C. (2007). Raman Spectroscopy Characterization of TiO_2_ Rutile Nanocrystals. Phys. Rev. B.

[47] Dias J.A., Freire A.L.F., Girotto I., Del Roveri C., Mastelaro V.R., Paris E.C., Giraldi T.R. (2021). Phase Evolution and Optical Properties of Nanometric Mn-Doped TiO_2_ Pigments. Mater. Today Commun..

[48] Saha M., Ghosh S., Paul S., Dalal B., De S.K. (2018). Nb-Dopant-Induced Tuning of Optical and Electrical Property of Anatase TiO_2_ Nanocrystals. ChemistrySelect.

[49] Vos K., Krusemeyer H.J. (1974). Low Temperature Electroreflectance of TiO_2_. Solid State Commun..

[50] Pankove J.I. (1975). Optical Processes in Semiconductors.

[51] Guang-Lei T., Hong-Bo H.E., Jian-Da S. (2005). Effect of Microstructure of TiO_2_ Thin Films on Optical Band Gap Energy. Chin. Phys. Lett..

[52] Mulmi D.D., Sekiya T., Kamiya N., Kurita S., Murakami Y., Kodaira T. (2004). Optical and Electric Properties of Nb-Doped Anatase TiO_2_ Single Crystal. J. Phys. Chem. Solids.

[53] Khomenko V.M., Langer K., Rager H., Fett A. (1998). Electronic Absorption by Ti^3+^ Ions and Electron Delocalization in Synthetic Blue Rutile. Phys. Chem. Miner..

[54] Bally A. (1999). Electronic Properties of Nano-Crystalline Titanium Dioxide Thin Films. EPFL.

[55] Nakamura I., Negishi N., Kutsuna S., Ihara T., Sugihara S., Takeuchi K. (2000). Role of Oxygen Vacancy in the Plasma-Treated TiO_2_ Photocatalyst with Visible Light Activity for NO Removal. J. Mol. Catal. A Chem..

[56] Prabhakarrao N., Rao T.S., Lakshmi K.V.D., Divya G., Jaishree G., Raju I.M., Alim S.A. (2021). Enhanced Photocatalytic Performance of Nb Doped TiO_2_/Reduced Graphene Oxide Nanocomposites over Rhodamine B Dye under Visible Light Illumination. Sustain. Environ. Res..

[57] Wang H.-Y., Chen J., Xiao F.-X., Zheng J., Liu B. (2016). Doping-Induced Structural Evolution from Rutile to Anatase: Formation of Nb-Doped Anatase TiO_2_ Nanosheets with High Photocatalytic Activity. J. Mater. Chem. A.

[58] Silva A., Muche D., Dey S., Hotza D., Castro R. (2015). Photocatalytic Nb_2_O_5_-Doped TiO_2_ Nanoparticles for Glazed Ceramic Tiles. Ceram. Int..

[59] Li X.D., Han X.J., Wang W.Y., Liu X.H., Wang Y., Liu X.R. (2012). Synthesis, Characterization and Photocatalytic Activity of Nb-Doped TiO_2_ Nanoparticles. Adv. Mater. Res..

[60] Gunawan P., Mei L., Teo J., Ma J., Highfield J., Li Q., Zhong Z. (2012). Ultrahigh Sensitivity of Au/1D α-Fe_2_O_3_ to Acetone and the Sensing Mechanism. Langmuir.

[61] Zaki M.I., Hasan M.A., Al-Sagheer F.A., Pasupulety L. (2000). Surface Chemistry of Acetone on Metal Oxides: IR Observation of Acetone Adsorption and Consequent Surface Reactions on Silica—Alumina versus Silica and Alumina. Langmuir.

[62] Zaki M.I., Hasan M.A., Pasupulety L. (2001). Surface Reactions of Acetone on Al_2_O_3_, TiO_2_, ZrO_2_, and CeO_2_: IR Spectroscopic Assessment of Impacts of the Surface Acid—Base Properties. Langmuir.

[63] Smith B.C. (2018). The C=O Bond, Part IV: Acid Anhydrides. Spectroscopy.

[64] Hadjiivanov K.I., Klissurski D.G. (1996). Surface Chemistry of Titania (Anatase) and Titania-Supported Catalysts. Chem. Soc. Rev..

[65] Hadjiivanov K. (2014). Identification and Characterization of Surface Hydroxyl Groups by Infrared Spectroscopy. Advances in Catalysis.

[66] Rumyantseva M.N., Makeeva E.A., Badalyan S.M., Zhukova A.A., Gaskov A.M. (2009). Nanocrystalline SnO_2_ and In_2_O_3_ as Materials for Gas Sensors: The Relationship between Microstructure and Oxygen Chemisorption. Thin Solid Films.

[67] Jeong N.C., Lee J.S., Tae E.L., Lee Y.J., Yoon K.B. (2008). Acidity Scale for Metal Oxides and Sanderson’s Electronegativities of Lanthanide Elements. Angew. Chemie Int. Ed..

