# Supplementary material for: Effect of Donor Nb(V) Doping on the Surface Reactivity, Electrical, Optical and Photocatalytic Properties of Nanocrystalline TiO2
Source: Materials (Basel). 2024 Jan 11;17(2):375. doi: 10.3390/ma17020375 (PMC10817237; doi:10.3390/ma17020375)
Supplement: Supplementary file 1 [file materials-17-00375-s001.zip › materials-2771460-supplementary.pdf]

Effect of Donor Nb(V) Doping on the Surface Reactivity, Electrical, Optical and Photocatalytic Properties of Nanocrystalline TiO_2

Dmitriy Kuranov [1,2], Anastasia Grebenkina [1], Alexandra Bogdanova [1], Vadim Platonov [1], Sergey Polomoshnov [2], Valeriy Krivetskiy [1,2] and Marina Rumyantseva [1*]

1 Chemistry Department, Lomonosov Moscow State University, 119991 Moscow, Russia
2 Scientific-Manufacturing Complex Technological Centre, 124498 Moscow, Russia
* Correspondence: roum@inorg.chem.msu

Supplementary Information

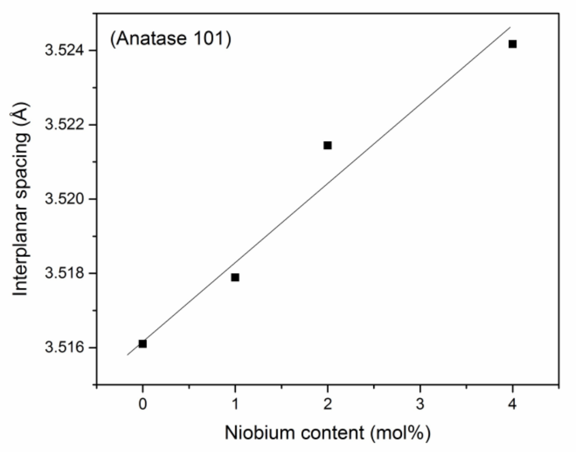

Figure S1a. The values of interplanar spaces for the family of planes 101 of TiO_2 anatase phase during doping with Nb(V).

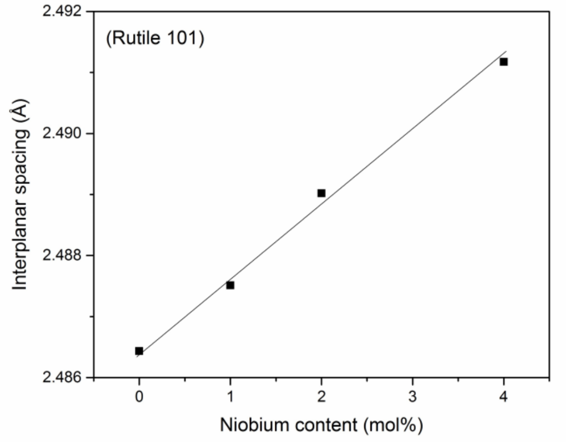

Figure S1b. The values of interplanar spaces for the family of planes 101 of TiO_2 rutile phase during doping with Nb(V).

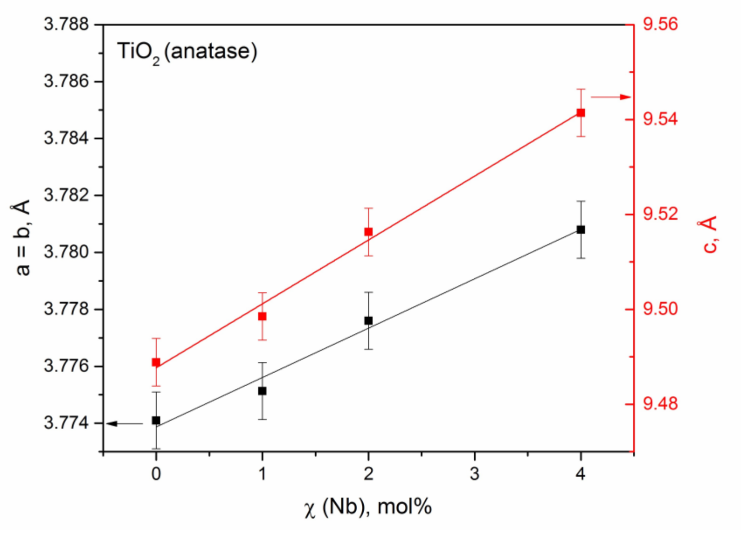

Figure S1c. The values of unit cell parameters of TiO_2 anatase phase during doping with Nb(V).

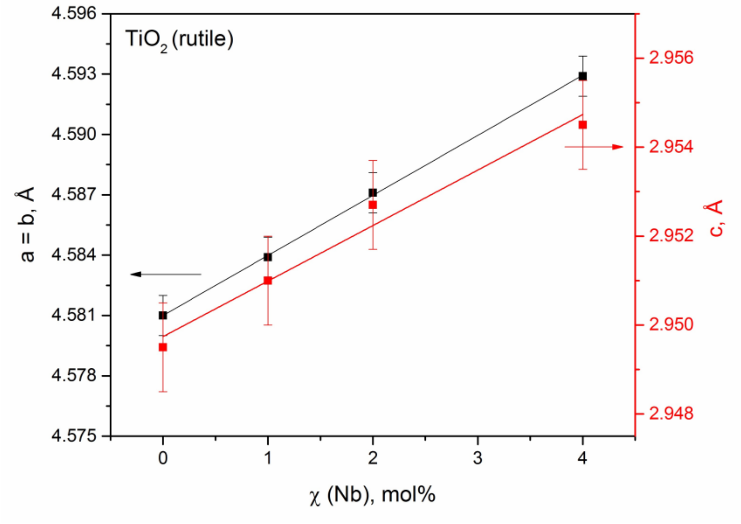

Figure S1d. The values of unit cell parameters of TiO_2 rutile phase during doping with Nb(V).

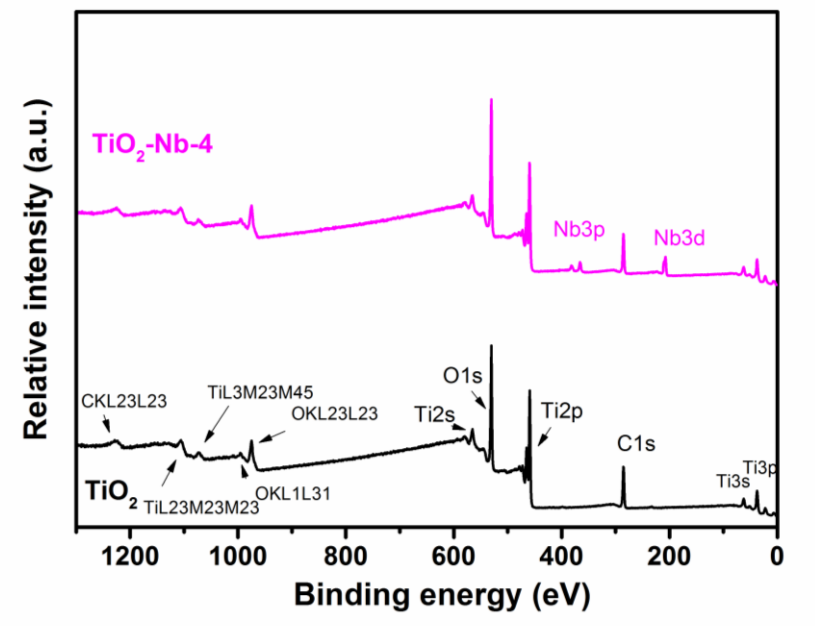

Figure S2. Review XP spectra of TiO_2 and TiO_2-Nb-4 materials.

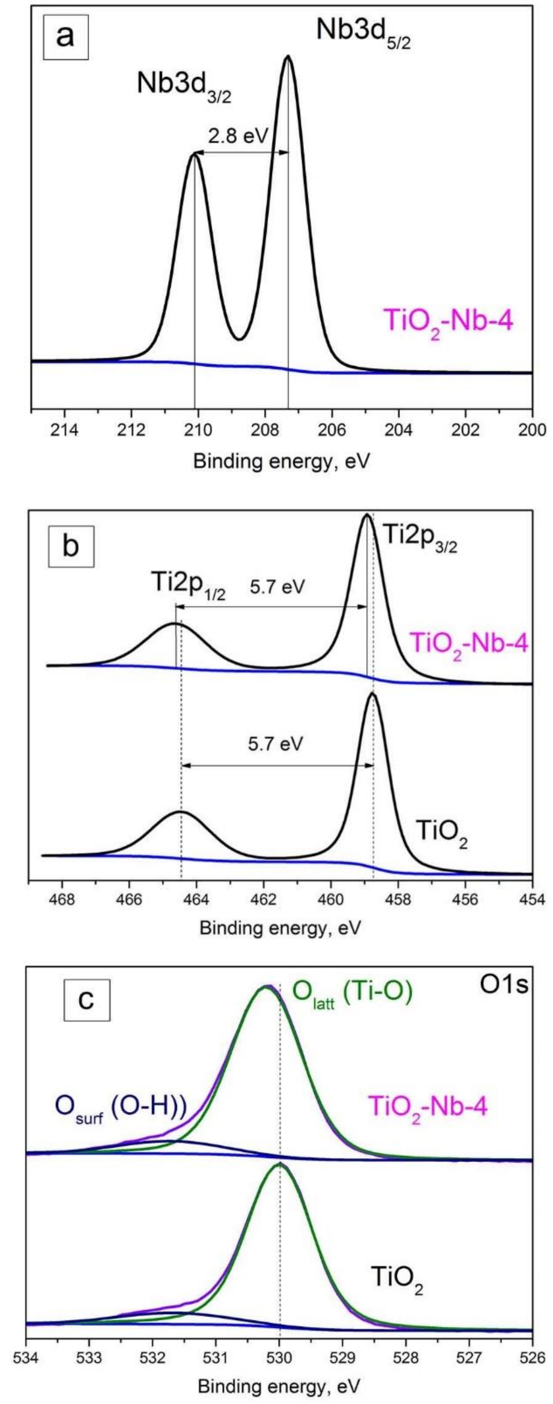

Figure S3a-c. Detailed XP spectra of TiO_2 materials.

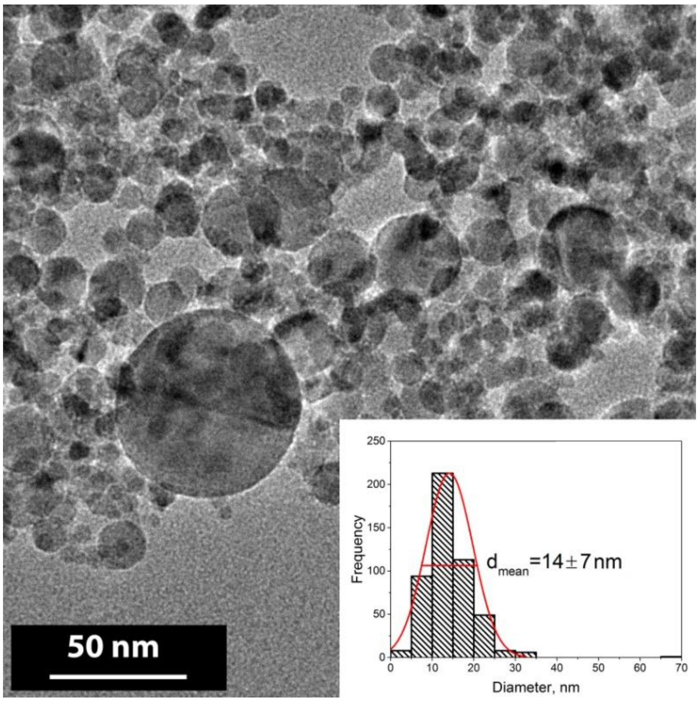

Figure S4. Bright field TEM photograph and histogram of normal distribution of TiO_2 pure material.

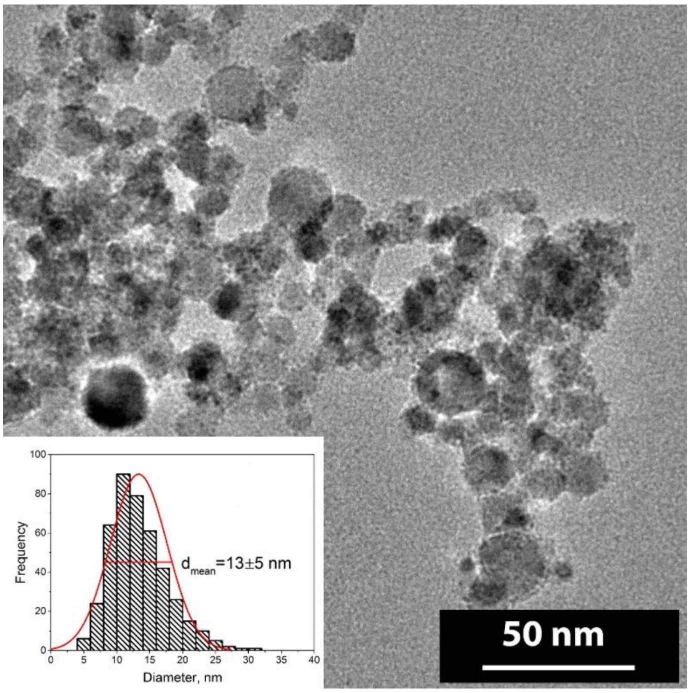

Figure S5. Bright field TEM photograph and histogram of normal distribution of TiO_2-Nb-4 material.

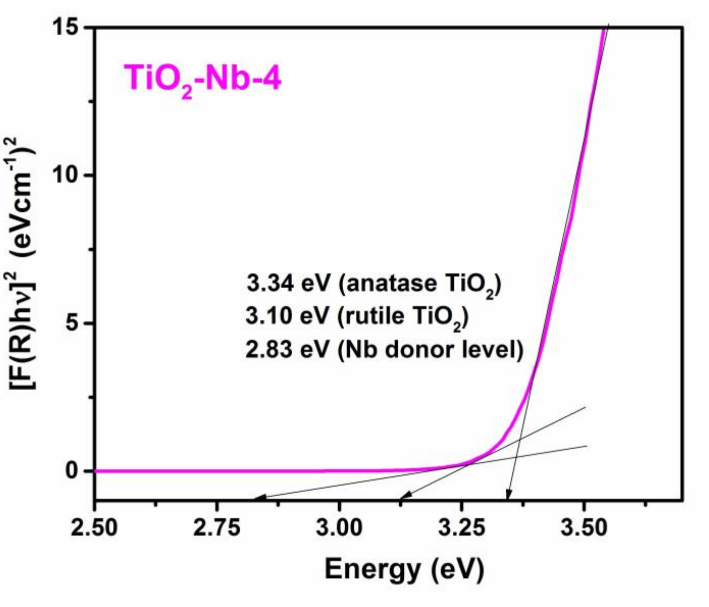

Figure S6. Tauc plot for TiO_2-Nb-4 materials (values of transitions energy are specified).

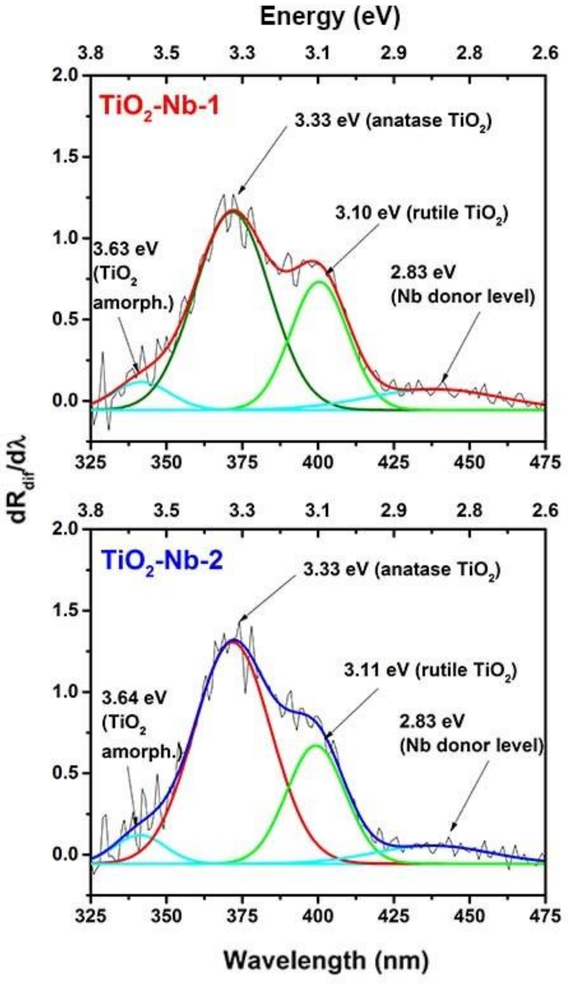

Figure S7. Differential diffuse reflectance spectra of materials TiO_2-Nb-1 and TiO_2-Nb-2.

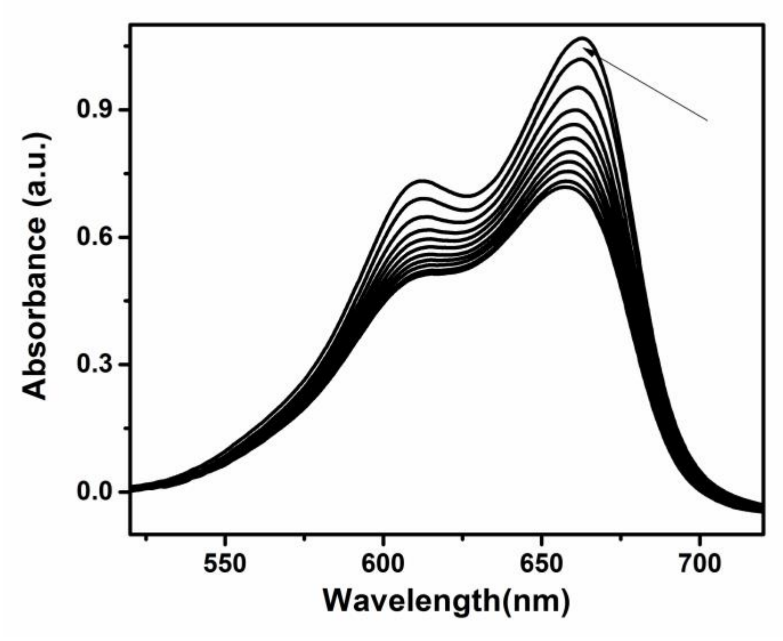

Figure S8. Absorption spectra of methylene blue solutions taken during photocatalysis.
